# A two-hop based adaptive routing protocol for real-time wireless sensor networks

**DOI:** 10.1186/s40064-016-2791-3

**Published:** 2016-07-19

**Authors:** Sandhya Rachamalla, Anitha Sheela Kancherla

**Affiliations:** Department of ECE, University College of Engineering, Osmania University, Hyderabad, India; Department of ECE, University College of Engineering, Jawaharlal Nehru Technological University, Hyderabad, India

**Keywords:** Deadline miss ratio, Energy consumption, End-to-end delay, Real-time routing, Adaptive transmission power

## Abstract

One of the most important and challenging issues in wireless sensor networks (WSNs) is to optimally manage the limited energy of nodes without degrading the routing efficiency. In this paper, we propose an energy-efficient adaptive routing mechanism for WSNs, which saves energy of nodes by removing the much delayed packets without degrading the real-time performance of the used routing protocol. It uses the adaptive transmission power algorithm which is based on the attenuation of the wireless link to improve the energy efficiency. The proposed routing mechanism can be associated with any geographic routing protocol and its performance is evaluated by integrating with the well known two-hop based real-time routing protocol, PATH and the resulting protocol is energy-efficient adaptive routing protocol (EE-ARP). The EE-ARP performs well in terms of energy consumption, deadline miss ratio, packet drop and end-to-end delay.

## Background

 Wireless sensor networks have gained attention of the research community in the recent years because of wide variety of applications that can be supported. Many WSN applications require real-time communication systems and examples of such applications can be found in many military, environment surveillance, disaster management and intelligent transportation systems (Chong and Kumar 2003). Among several aspects of WSNs, energy conservation and delay, supporting quality of service (QoS) in WSNs is still a largely unexplored research field (Chen et al. 2006).

Although energy efficiency is the primary concern in WSNs for longer network lifetime, the low latency communication is gaining more importance in new applications. Out-of-date information will be irrelevant, mainly in real-time environments and may lead to negative effects to the systems (Li et al. 2009).

Timeliness is important in sending crucial messages in industrial WSNs. And sensor nodes are battery operated for energy supply. Hence energy efficiency and latency are the important design goals in WSNs. Supporting real-time QoS in WSNs can be addressed from different layers and mechanisms (Li et al. 2008). Cross-layer optimization can provide further improvement and above all, routing protocol plays a crucial role in supporting end-to-end QoS (Li et al. 2009). Here, in this paper, the focus is on the timely delivery of packets within deadline and end-to-end QoS i.e. the messages are to be transmitted in time to take prompt actions and energy efficiency.

To reduce the complexity of the systems, most of routing protocols are based on one-hop neighbourhood information (Li et al. 2007). However, multi-hop information based routing can perform better as more information about the neighbours of a node in the network is available and that is effectively utilized in broadcast operations, channel access methods etc. (Shue Chen et al. 2008; Spohn et al. 2004; Calinescu 2003).

It is observed from the study that two-hop based routing results in less number of hops from source to sink when compared with that of one-hop based routing (Shue Chen et al. 2008). However, it is not attractive to go for three-hop based routing, as it further increases the complexity which may not be affordable for the given network application. Hence in this paper, the proposed routing mechanism is integrated with PATH, the well-known two-hop based real-time routing algorithm for WSNs.

In this paper, an efficient routing mechanism is proposed with the following goals:

- Save more energy of nodes by removing early all much delayed packets or useless packets according to their residual deadline requirements and expected end-to-end delay (E2E delay) as in Aissani et al. (2013).
- Adjust the transmission power based on the attenuation of the wireless link (Rodoplu and Meng 1999) without degrading the real-time flow of packets. It further results in effective utilization of energy.
- To improve the routing performance in terms of real-time QoS with two-hop neighbourhood information, with the association of PATH.

In our network model, the following assumptions are made.

- WSN consists of (N − 1) nodes and a sink, all of which are randomly distributed in the field.
- The nodes are aware of their positions with respect to sink.
- Each node needs to know its immediate and two-hop neighboring nodes current status, i.e. their IDs, positions, residual energies etc. This can be done with two-rounds of HELLO messages’ broadcasting.
- All the nodes are powered by a non renewable energy source i.e. when this energy supply is exhausted the sensors becomes non-operational.
- Nodes are assumed to be stationary else additional HELLO messages are required to update neighborhood information.
- All the nodes are aware of their residual energy and have same transmission range and transmission rate.
- All nodes share the same wireless medium. The sensors are neighbors if they are in the transmission range of each other and can directly communicate with each other.
- The network density is assumed to be enough to prevent void situation.
- The bandwidth of each link between the nodes is assumed to be constant.

The rest of the paper is organized as follows. “Real-time routing protocols for WSNs” section summarizes related routing protocols and their performance measures. “Design of EE-ARP for RT-WSN” section presents our proposed mechanism which aims to improve energy consumption and real-time QoS. The performance of the proposed protocol is evaluated in “Performance evaluation” section. Simulations and comparisons are discussed in this section. “Conclusion and future scope” section concludes the paper and possible enhancements are discussed.

## Real-time routing protocols for WSNs

Many researchers have provided solutions for real-time routing in WSNs. This section provides the analysis of the various existing real time routing protocols for WSNs emphasizing their strengths and weaknesses and various other challenges. Real time routing is discovering an optimum route from source to destination which meets the real time constraints. Timely and reliable data delivery is very important for positive results as out-dated data may lead to disaster effects.

AODV (Perkins and Royer 1999) is an on-demand routing protocol which builds route between the nodes with sequence numbers, only when the source node demands for routing the sensed data. But the intermediate nodes can lead to inconsistent routes if the sequence number is old when no route exists for a new destination; a *route discovery* process is invoked, which leads to the significant delays in sensor networks. DSR (Johnson et al. 1996) protocol is another on-demand routing protocol which eliminates the periodic updating of routing tables as it is *beacon*-*less.* In case of broken link, the source node finds the new route only after receiving the *RouteError* from the node adjacent to the broken link. RAP (Lu et al. 2002) is the first real-time communication architecture that handles the deadline issues pertaining to large scale WSNs. It uses the high level ‘query and event’ services and the *velocity monotonic scheduling* (VMS) policy to schedule packets.

SPEED (He et al. 2003) protocol is an important real-time communication protocol to route packets with the desired speed for sensor networks. It doesn’t consider the energy metric and single speed is considered. MMSPEED (Felemban et al. 2006) extends the SPEED protocol to support different velocities and level of reliability for multiple probabilistic QoS guarantee in WSNs. The QoS provisioning is performed in two quality domains, namely *timeliness* and *reliability*. RPAR (Chipura et al. 2006) is the advance version of RAP. It is the first protocol that is designed to support the real time routing for WSNs with power control. Application specific communication delays are handled in this protocol by dynamically adapting transmission power and routing decisions based on the workload and packet deadlines. The proposed power adaption and neighbourhood mechanisms are on-demand and hence this protocol is a reactive protocol.

THVR (Li et al. 2009) is a two-hop neighbourhood information-based routing protocol for real time wireless sensor networks proposed to support the QoS in terms of real-time packet delivery along with better energy efficiency. PATH (Rezayat et al. 2010) is another real-time protocol which uses the two-hop neighbour information for routing decisions. Dynamic adjustment of transmission power is adopted to reduce the probability of packet dropping. It provides the service differentiation and serves different data traffic using *dynamic velocity assignment* and control trade-off between energy and delay constraint with *dynamic power control.* Hence, packet dropping is reduced with dynamic performance improving the real-time routing in WSNs.

JiTS (Liu et al. 2006) shows that shortest path routing provides better performance than geographic routing and explores several policies for non-uniformly delaying data at several intermediate nodes for contention-free transmission. EARTOR (Yang et al. 2010) aims to maximize the number of requests in the network and route requests are designed with specified latency constraints. Cross layer design is adopted in EARTOR along with the mechanism for each relay node that takes into consideration residual energy, location information and relay node priority. EEOR (Mao et al. 2011) improves the throughput by allowing nodes that overhear the transmission to participate in forwarding the packet. The nodes are prioritized and low priority forwarder will discard the packet if the packet has been forwarded by high priority forwarder. The selection of forwarder list and prioritizing is a challenging task in it to have optimized energy consumption. Satisfying the desired link reliability along with two-hop routing is proposed in (Shiva Prakash et al. 2013). It needs the calculation of two-hop velocity, delay estimation and node forwarding metric based on reliability, velocity and energy.

In our proposed mechanism, we adopt the approach of identifying the slow packets, which are useless and cannot meet the prescribed deadline, and remove those packets from the queue of intermediate nodes located near to the sink. This conserves energy and improves the network lifetime. It also implements the adaptive transmission power algorithm, which dynamically changes the transmission power to be used in forwarding metric, instead of fixed transmission power as in THVR. Though power adaption scheme is used in PATH, it is invoked when it cannot find suitable two-hop neighbour and when it has sufficient choice of forwarding pair. During transmission, the power is adjusted according to the location of the receiver and the quality of the wireless link. This further improves energy efficiency. EE-ARP implements the same forwarding metric as used in PATH, finding the suitable next forwarding pair based on the novel two-hop velocity integrated with energy balancing mechanism which maintains the routing efficiency without degrading the real-time behaviour of the protocol. We refer to it as energy-efficient adaptive routing protocol (EE-ARP). The proposed routing protocol details are given in the next section.

## Design of EE-ARP for RT-WSN

The proposed protocol route the packets in three stages: (1) identification and removal of much delayed packets, (2) adaptive transmission power algorithm and (3) forwarding metric.

### Identification and removal of much delayed packets

Not all the packets routed for transmission have the chance to reach their destination because of insufficient deadline. The deadline information is exploited in the proposed routing protocol and the much delayed packets or slow packets are removed from the queue of intermediate nodes near the sink as it is useless to traverse those packets towards destination, thereby saving the energy of nodes. The queue is now maintained to have only packets with sufficient residual deadline. To identify the slow packet from the queue, EE-ARP calculates the expected delay for the current packet to reach the destination and decides whether to remove or not, the current packet based on this expected delay.

1. *Expected delay* The expected delay for the current packet *p* at the current node *x* to reach the destination *d* is *Txd*(*p*) and is given by the formula (1).

1 $$Txd(p) = \frac{Dxd(p)}{Dsx(p)} \times Tsx(p)$$

**Fig. 1:**
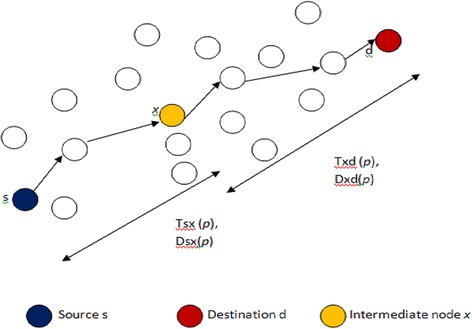
Expected delay estimation

As shown in Fig. 1, *Dxd*(*p*) denotes the remaining geographic distance that the current packet *p* from current node *x* to the destination *d*, *Dsx*(*p*) is the geographic distance travelled by the packet *p* from source *s* to current node *x*. *Tsx*(*p*) gives the delay for the packet to reach to the current node *x*.

**Fig. 2:**
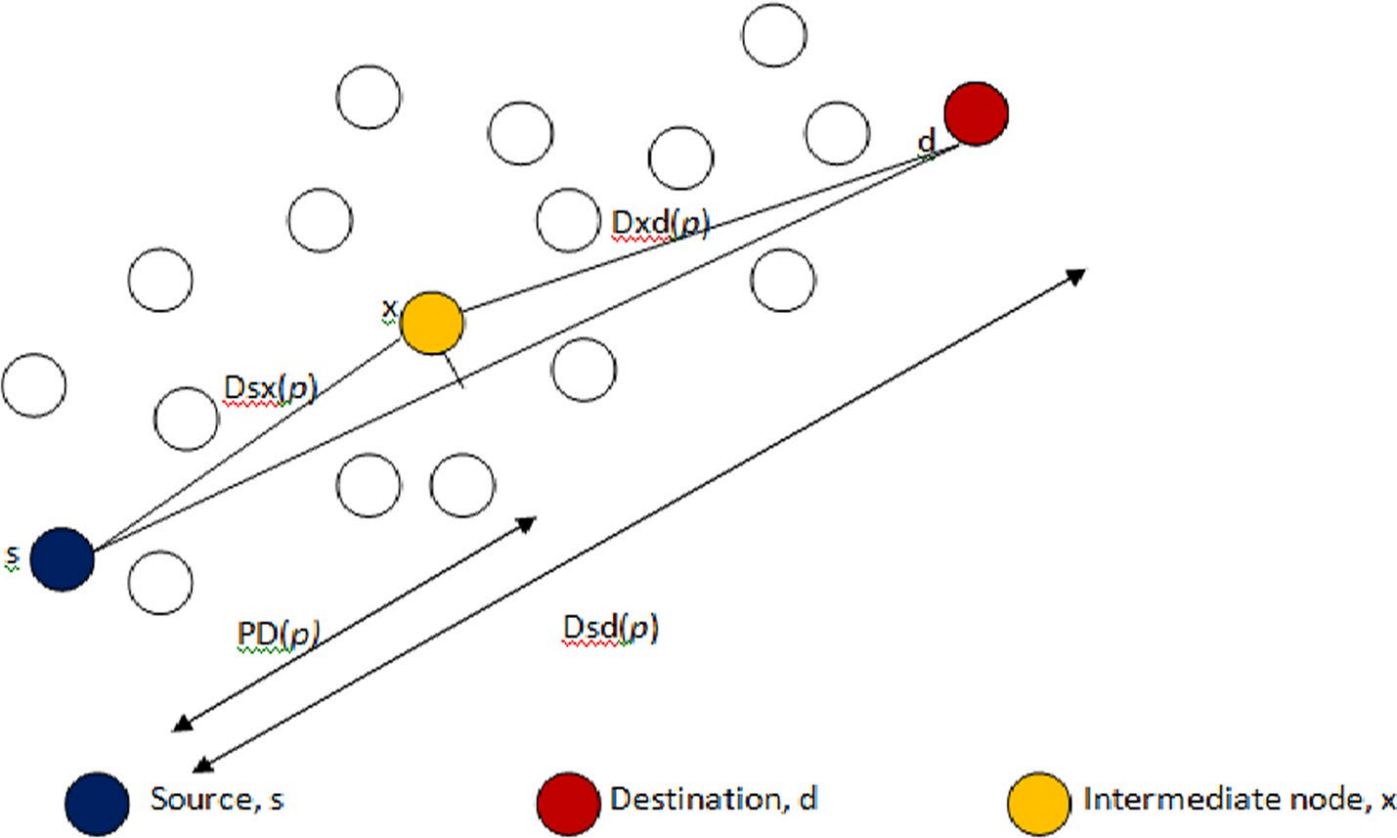
Illustration of progressive distance *PD*(*p*)

2. *Removal of much delayed packets* After having the expected delay for the current packet *p* at current node *x*, it is to be decided whether the packet can be retained or not in the queue of intermediate node. The distance between source *s* and destination *d*, *Dsd*(*p*) and progressive distance *PD*(*p*), the distance that the packet *p* progressed towards the destination, are used in the decision rule. Figure 2 shows the *PD*(*p*). The complete algorithm for the *identification and removal of slow packets* at each intermediate node is given in Algorithm 1.

**Fig. 3:**
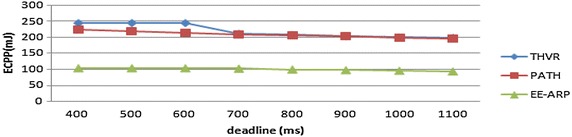
ECPP comparison of THVR, PATH and EE-ARP

**Fig. 4:**
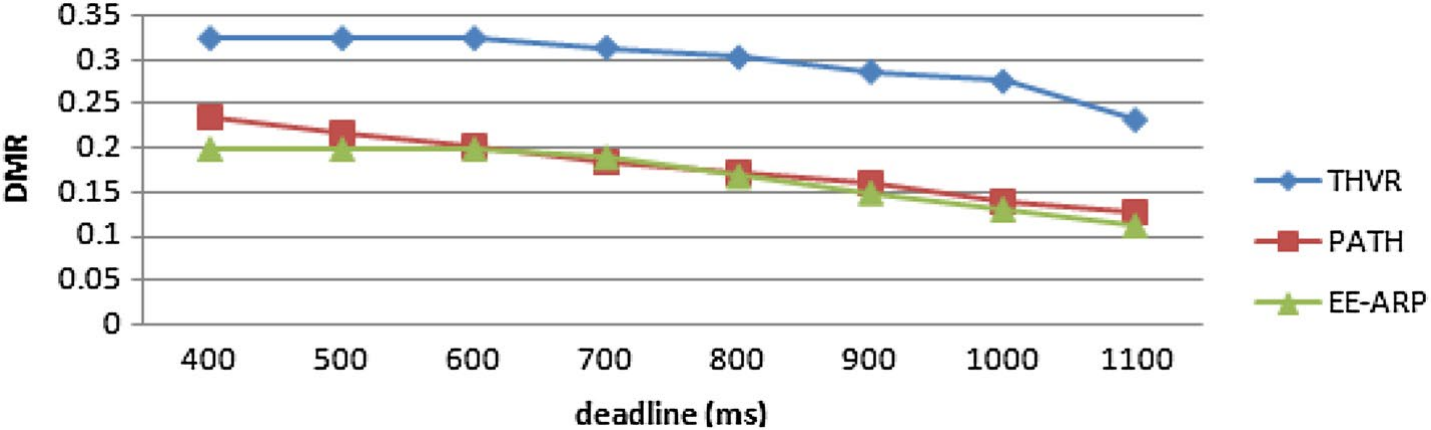
DMR comparison of THVR, PATH and EE-ARP

**Fig. 5:**
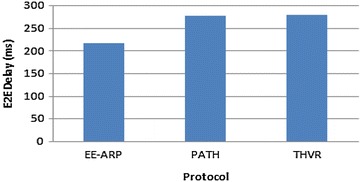
E2E delay comparison for the protocols THVR, PATH and EE-ARP

The Algorithm 1 is as follows:

**Table:** **Algorithm 1: Identification and removal of slow packets**

1. **For** each current packet *p* at the current node *x*,
2. **Calculate** expected delay *Txd*(*p*);
**#** ***Txd***(***p***) is the expected delay for the packet p to reach the remaining distance until destination d.
**#** ***PD***(***p***) is the distance that the packet p progressed towards the destination d.
**#** ***Dsd*** *(* ***p*** *)* is the distance between source s and destination d.
**#** **α** is the parameter chosen according to the application, it must be close to 1 for real-time applications and close to 0 for energy-efficient applications.
**# Packet removal decision rule.**
3. **If** *PD*(*p*) ˃ α × *Dsd*(*p*) **then**
4. **If** *Txd*(*p*) ˃ deadline (*p*) **then**
5. **Remove** packet *p* from the queue of current node *x*
6. **Endif**
7. **Else**
8. **If** $\frac{PD(p)}{\alpha \times Dsd(p)} \times Txd(p)$ ˃ deadline (*p*) **then**
9. **Remove** packet *p* from the queue of current node *x*
10. **Endif**
11. **Endif**

The *PD*(*p*) is calculated as shown in formula 2.

2 $$PD(p) = \left\{{\begin{array}{*{20}l} {\frac{{D^{2} sx(p) - D^{2} xd(p) + D^{2 } sd(p)}}{2 \times Dsd(p)}} \hfill &\quad {if\,Dsx(p) < Dsd(p)} \hfill \\ {Dsd(p)} \hfill &\quad {otherwise} \hfill \\ \end{array}} \right.$$

The Algorithm 1 explains the procedure to identify and remove the unwanted slow packets from reaching the destination because of insufficient deadline and to preserve energy of the nodes by not forwarding them towards the destination. After calculating the expected delay as shown in formula 1, the packet removal decision rule is applied as shown in Algorithm 1. During simulation, the parameter α is chosen 0.5. So, the packet is tested only when it is progressed more than 50 % of the total distance. i.e. if *PD*(*p*) is >0.5 × *Dsd* (*p*), then the expected delay for the packet *p*, *Txd*(*p*), is compared with the required deadline, deadline (*p*), which is set according to the application requirements. If the packet *p* cannot meet the deadline requirement then it is removed from the queue of the current or intermediate node *x*. Otherwise, more chance is given to the packet *p* to reach the destination with *Txd*(*p*) multiplied with $\frac{PD(p)}{\alpha \times Dsd(p)}$ and compared with given deadline. If the value exceeds the deadline, then the packet is removed.

### Adaptive transmission power algorithm

The queue of the current node now contains the useful packets after the removal of useless packets. The transmission power of each packet is adaptively varied based on the quality of wireless link and this power is used in forwarding metric for choosing the next candidate. In path loss model, the transmit power falls as 1/*d*^*n*^, where *d* is the distance between the sender and receiver and *n* is the path loss exponent, this idea is exploited in the proposed routing mechanism. The remaining energy is only considered in THVR in forwarding metric. In PATH, both the forwarding energy and remaining energy are considered. The adaptive transmission power algorithm is described as follows.

**Table:** **Algorithm 2: Adaptive transmission power algorithm**

1. While forwarding a packet *p* in a queue of intermediate node *x*, the transmission power *P*(*x*) is given by formula (3)
$$P\left( x \right) = t.d^{n } + C\quad \quad \quad (3)$$
# ***d*** is the distance from current node to the next forwarding node.
#***n*** is the path loss exponent and depends on the quality of wireless link (*n* ≥ 2).
#***C*** is the system processing cost and *t* is prediction threshold.
2. The quantity of energy required to send a packet is proportional to the transmission power of the current node. The transmission energy *E(x)* is given
$$E(x) = P(x) \times T(x)\quad \quad \quad (4)$$
# ***T***(***x***) is the transmission time, the time required to send a packet by a node.

In the proposed routing protocol, the transmission power is varied based on the geographic position of next choice and is useful in saving the energy instead of fixed transmission power as used in THVR. In PATH, the power adaptation scheme is used but it is invoked only when there is no suitable forward choice or when more than one forwarding choice exists. During simulation, the path loss exponent *n* is chosen to be 2 and system processing cost *C* is assumed to be 0.

### Forwarding metric

The forwarding metric used in the proposed protocol utilizes the two-hop neighbourhood information of the network as in THVR and PATH, which improves the routing performance when compared with that of one-hop neighbourhood information and the same forwarding metric, which is based on velocity and energy metric, is used to select next forwarding pair for the packet *p* to get routed towards destination, as in PATH. But the transmission energies are adaptively calculated, as shown in formula 4, based on the distance between sender and receiver. This improves the energy efficiency and better forwarding pairs are selected in routing the packets.

## Performance evaluation

**Fig. 6:**
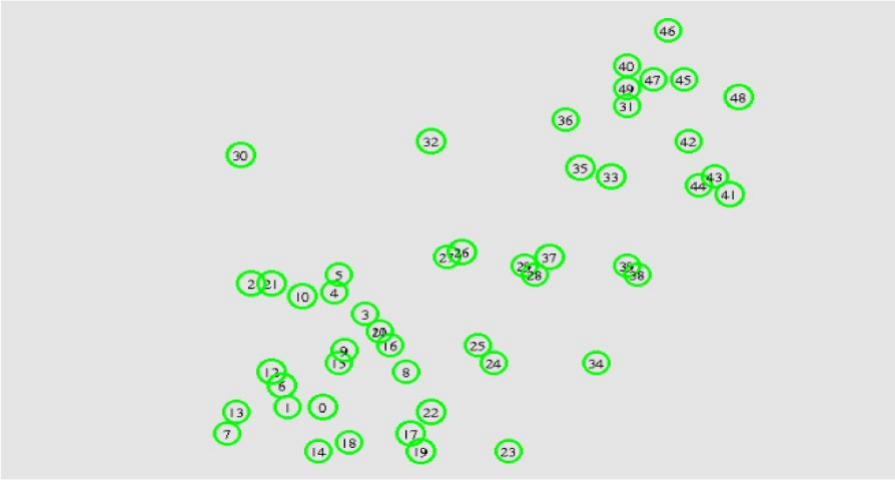
Wireless sensor network model with random distribution of nodes

The proposed routing protocol EE-ARP is simulated in Network Simulator. The Network simulator is used to simulate TCP, routing and multicast protocols over wired/wireless networks, from application to communication layers. It provides simple and realistic radio propagation and MAC models. We set the parameters close to practical WSN according to MicaZ motes with MPR2400 (2.4 GHz) radio. These motes are used for enabling low-power, wireless sensor networks with globally compatible ISM band (2.4–2.48 GHz). Nodes are randomly distributed in a 500 m × 500 m area as shown in Fig. 6. We considered one source and one destination. The source node (ID 1) is chosen at the left-lower corner of the sensing area fixed at the location (95, 50 m), while the destination node (ID 46) is fixed at the location (430, 475 m) at right-top corner of the sensing area. The proposed protocol EE-ARP is investigated and compared with THVR and PATH protocols. The source generates CBR traffic at 10 kbps rate with packet frame size 64 bytes (including header and CRC fields).

The performance metrics are (1) energy consumption per packet (ECPP), which is defined by the total energy expended divided by the number of successfully transmitted packets, (2) deadline miss ratio (DMR), which is defined as the ratio of packets that miss the predefined deadline, (3) packet drop and (4) E2E delay. The proposed protocol is compared with two-hop based routing protocols THVR and PATH for the same network scenario and simulation settings. The deadline requirement is varied from 400 to 1100 ms and in each run DMR is calculated for the three protocols. The results show that the proposed protocol offers better energy efficiency than the other two protocols as shown in Fig. 3. This is due to the adaptive transmission power algorithm and the novel method of removal of slow packets thereby saving the energy of nodes in the network.

The DMR is also improved in EE-ARP as shown in Fig. 4, because of the removal of much delayed packets at intermediate nodes and preventing them to reach the destination with large delay. In THVR and PATH, the packets are given chance to progress and initiative drop controller is invoked to decide whether a packet should be drop or not. In EE-ARP, the drop controller is not used as in PATH and THVR. Instead the novel method of removal of much delayed packets is employed. This method helps in the removal of slow packets from the queue and only the packets which have sufficient residual deadline are retained for routing. Also the efficient utilization of energy results in better forwarding choice and the packets are routed effectively which further reduces DMR. The average E2E delay of all the packets is also less in EE-ARP, as shown in Fig. 5, as slow packets are removed and only useful packets with sufficient deadline are allowed to reach the sink.

**Fig. 7:**
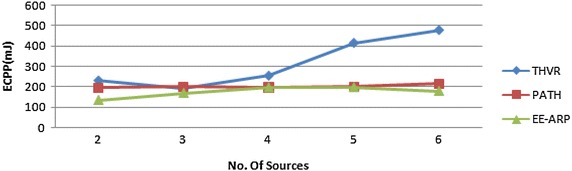
Performance of ECPP with multiple sources

**Fig. 8:**
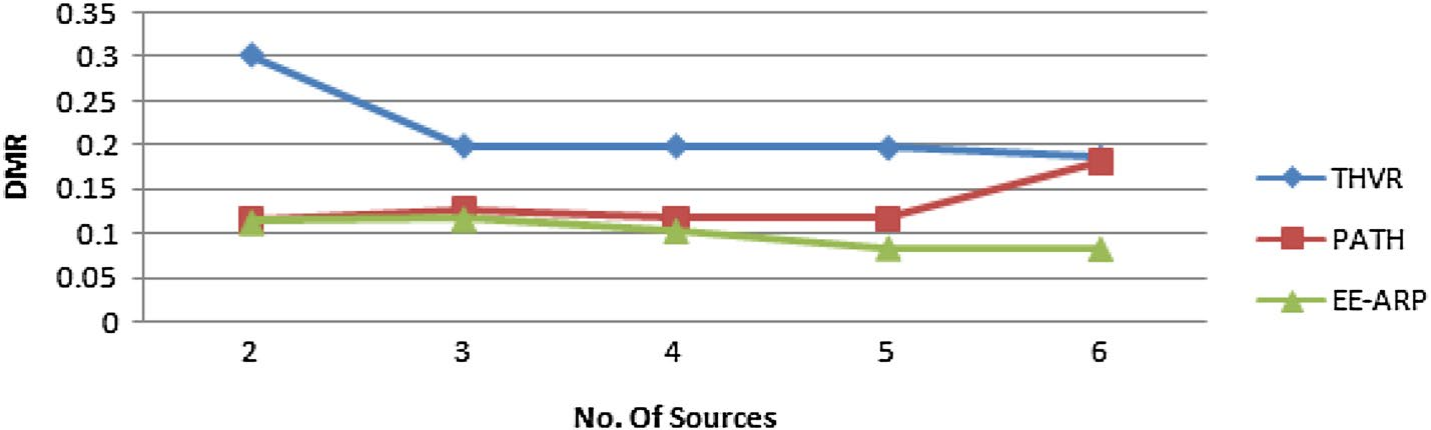
Performance of DMR with multiple sources

Furthermore, we investigate the performance of EE-ARP with multiple sources. Figure 7 shows the ECPP for the three protocols, THVR, PATH and EE-ARP, in which the number of sources increases from 2 to 6, while the deadline is fixed at 400 ms. Each source generates CBR traffic at 10 kbps rate with each packet frame size 64 bytes (including header and CRC fields). On the other hand, Fig. 8 shows the DMR performance of three protocols. The source nodes are located in bottom left area and labelled with ID {1, 6, 7, 13, 14, 18}, as shown in Fig. 6, respectively.

As the number of sources increases, the energy consumption and DMR generally increases, which is due to the increase in workload and traffic congestion. The comparison shows that EE-ARP has low ECPP and DMR due to the novel method of removing the useless packets and adaptive transmission power algorithm. When number of sources increases, the network congestion increases resulting in decrease in the probability of channel access, thereby the increasing collision of packets at MAC layer.

**Fig. 9:**
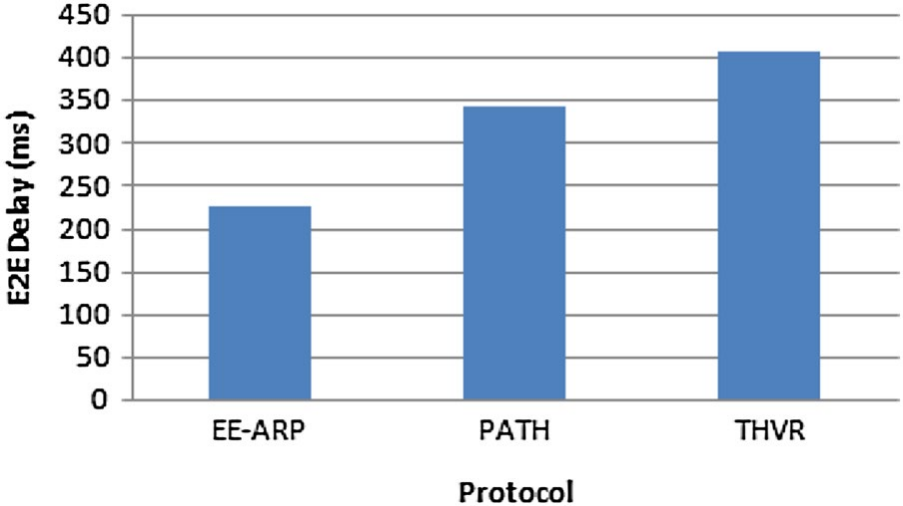
E2E delay comparison when no of sources = 5

Figure 9 gives the E2E delay performance of the three protocols and it shows that the proposed mechanism gives less E2E delay as slow packets are removed effectively and due to the conservation of nodes’ energy, the latency is low and results in better routing of packets with velocity-energy based forwarding metric.

**Fig. 10:**
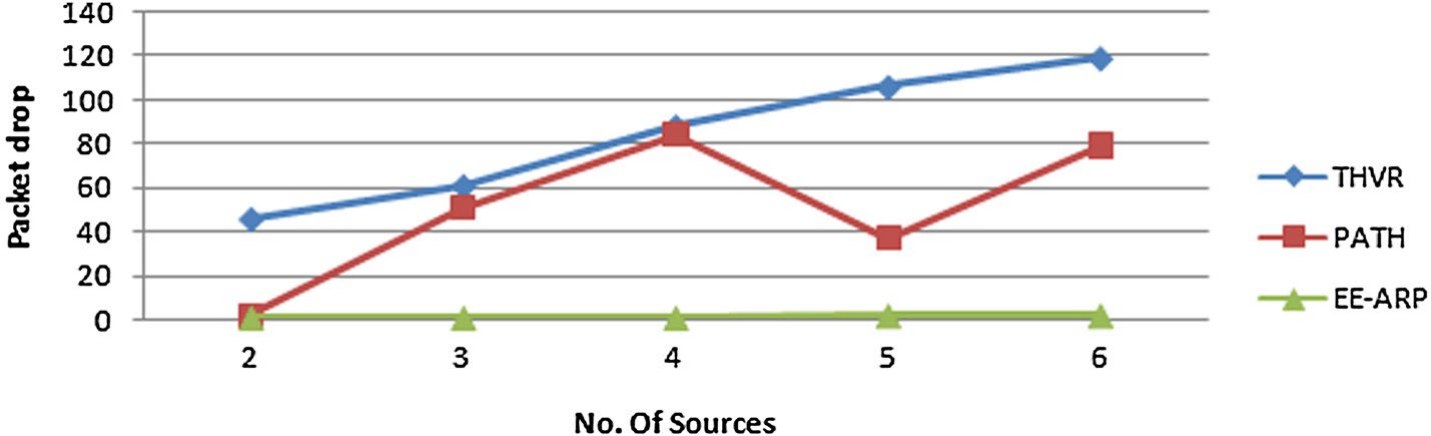
Packet drop for THVR, PATH and EE-ARP

From Fig. 10, it is observed that the packet drop is lowest in EE-ARP, as the packets with sufficient deadline are forwarded (removing useless packets from queue) towards destination, hence congestion is less. Moreover, the adaptive transmission power algorithm ensures energy efficiency, better routing performance and increased network lifetime. The less latency and efficient energy utilization results in fewer packets drop in the proposed mechanism.

## Conclusion and future scope

In this paper, an adaptive routing protocol based on two-hop neighbourhood information of the network is proposed. It is integrated with a novel real-time power aware two-hop based protocol PATH. It employs a novel method of removal of much delayed packets and also the efficient adaptive transmission power algorithm to achieve better energy efficiency without degrading the real-time performance in WSNs. This integration reduces the energy consumption and improves DMR better than THVR and PATH. We have investigated for multiple sources in the network thereby increasing the traffic intensity and the performance of the proposed protocol is observed in terms of DMR, ECPP, E2E delay and packet drop. EE-ARP outperforms THVR and PATH.
